# Supplementary material for: Simultaneous inhibition of bacterial virulence and anti-phage defense systems by synergistic bacteriophage counter-defense proteins
Source: EMBO J. 2026 Mar 18;45(8):2756–84. doi: 10.1038/s44318-026-00740-0 (PMC13083879; doi:10.1038/s44318-026-00740-0)
Supplement: Supplementary file 1 — Table EV1 [file 44318_2026_740_MOESM1_ESM.docx]

Table EV1. Strains, plasmids and phages used in this study

| **Strain or plasmid** | **Relevant characteristics** | **Source** |
| --- | --- | --- |
| ***P. aeruginosa*** |  |  |
| PAO1 | Reference strain, derivative of PAO | (Liang et al., 2008) |
| Δ*exsA* | *exsA* knockout mutant of PAO1; Gm^r^ | (Kong et al., 2013) |
| Δ*lon* | *lon* deletion mutant of PAO1 | (Yang et al., 2015) |
| Δ*lon*/*p-lon* | *lon* complement strain，  derived from Δ*lon* and pAK1900-*lon*; Cb^r^ | (Yang et al., 2015) |
| PAO1/pME6032-*dap2* | Overexpression of *orf004* using pME6032 plasmid in PAO1 | This study |
| PAO1/pME6032-*dap2*(V52KA64KN79A) | Overexpression of *dap2*(V52KA64KN79A) using pME6032 plasmid in PAO1 | This study |
| PAO1/pME6032*-orf001* | Overexpression of *orf001* using pME6032 plasmid in PAO1 | This lab |
| PAO1/pME6032*-orf003* | Overexpression of *orf003* using pME6032 plasmid in PAO1 | This lab |
| PAO1/pME6032*-orf005* | Overexpression of *orf005* using pME6032 plasmid in PAO1 | This lab |
| PAO1/pME6032*-orf006* | Overexpression of *orf006* using pME6032 plasmid in PAO1 | This lab |
| PAO1/pME6032*-orf007* | Overexpression of *orf007* using pME6032 plasmid in PAO1 | This lab |
| PAO1/pME6032*-orf008* | Overexpression of *orf008* using pME6032 plasmid in PAO1 | This lab |
| PAO1/pME6032*-orf009* | Overexpression of *orf009* using pME6032 plasmid in PAO1 | This lab |
| PAO1/pME6032*-orf010* | Overexpression of *orf010* using pME6032 plasmid in PAO1 | This lab |
| PAO1/pME6032*-orf011* | Overexpression of *orf011* using pME6032 plasmid in PAO1 | This lab |
| PAO1/pME6032*-orf012* | Overexpression of *orf012* using pME6032 plasmid in PAO1 | This lab |
| PAO1/pME6032*-orf013* | Overexpression of *orf013* using pME6032 plasmid in PAO1 | This lab |
| PAO1/pME6032*-orf131* | Overexpression of *orf131* using pME6032 plasmid in PAO1 | This lab |
| PAO1/pME6032*-orf132* | Overexpression of *orf132* using pME6032 plasmid in PAO1 | This lab |
| PAO1/pME6032*-orf133* | Overexpression of *orf133* using pME6032 plasmid in PAO1 | This lab |
| PAO1/pME6032*-orf134* | Overexpression of *orf134* using pME6032 plasmid in PAO1 | This lab |
| PAO1/pME6032*-orf135* | Overexpression of *orf135* using pME6032 plasmid in PAO1 | This lab |
| PAO1/pME6032*-orf136* | Overexpression of *orf136* using pME6032 plasmid in PAO1 | This lab |
| PAO1/pME6032*-orf137* | Overexpression of *orf137* using pME6032 plasmid in PAO1 | This lab |
| PAO1/pME6032*-orf138* | Overexpression of *orf138* using pME6032 plasmid in PAO1 | This lab |
| PAO1/pME6032*-orf139* | Overexpression of *orf139* using pME6032 plasmid in PAO1 | This lab |
| PAO1/pME6032*-orf140* | Overexpression of *orf140* using pME6032 plasmid in PAO1 | This lab |
| PAO1/pME6032*-orf141* | Overexpression of *orf141* using pME6032 plasmid in PAO1 | This lab |
| PAO1/pME6032*-orf142* | Overexpression of *orf142* using pME6032 plasmid in PAO1 | This lab |
| PAO1/pME6032*-orf143* | Overexpression of *orf143* using pME6032 plasmid in PAO1 | This lab |
| PAO1/pME6032*-orf144* | Overexpression of *orf144* using pME6032 plasmid in PAO1 | This lab |
| PAO1/pME6032*-orf145* | Overexpression of *orf145* using pME6032 plasmid in PAO1 | This lab |
| PAO1/pME6032*-orf146* | Overexpression of *orf146* using pME6032 plasmid in PAO1 | This lab |
| PAO1/pME6032*-orf147* | Overexpression of *orf147* using pME6032 plasmid in PAO1 | This lab |
| PAO1/pME6032*-orf148* | Overexpression of *orf148* using pME6032 plasmid in PAO1 | This lab |
| PAO1/pME6032*-orf149* | Overexpression of *orf149* using pME6032 plasmid in PAO1 | This lab |
| PAO1/pME6032*-orf150* | Overexpression of *orf150* using pME6032 plasmid in PAO1 | This lab |
| PAO1/pME6032-*orf151* | Overexpression of *orf151* using pME6032 plasmid in PAO1 | This lab |
| PAO1/pME6032*-orf152* | Overexpression of *orf152* using pME6032 plasmid in PAO1 | This lab |
| PAO1/pME6032*-orf153* | Overexpression of *orf153* using pME6032 plasmid in PAO1 | This lab |
| PAO1/pME6032*-orf154* | Overexpression of *orf154* using pME6032 plasmid in PAO1 | This lab |
| PAO1/pME6032*-orf155* | Overexpression of *orf155* using pME6032 plasmid in PAO1 | This lab |
| PAO1/pME6032*-orf156* | Overexpression of *orf156* using pME6032 plasmid in PAO1 | This lab |
| PAO1/pME6032*-orf158* | Overexpression of *orf158* using pME6032 plasmid in PAO1 | This lab |
| PAO1/pME6032*-orf161* | Overexpression of *orf161* using pME6032 plasmid in PAO1 | This lab |
| PAO1/pME6032*-orf162* | Overexpression of *orf162* using pME6032 plasmid in PAO1 | This lab |
| PAO1/pME6032*-orf166* | Overexpression of *orf166* using pME6032 plasmid in PAO1 | This lab |
| PAO1/pME6032*-orf167* | Overexpression of *orf167* using pME6032 plasmid in PAO1 | This lab |
| PAO1/pME6032*-orf168* | Overexpression of *orf168* using pME6032 plasmid in PAO1 | This lab |
| PAO1/pME6032*-orf169* | Overexpression of *orf169* using pME6032 plasmid in PAO1 | This lab |
| PAO1/pME6032*-orf170* | Overexpression of *orf170* using pME6032 plasmid in PAO1 | This lab |
| PAO1/pME6032*-orf171* | Overexpression of *orf171* using pME6032 plasmid in PAO1 | This lab |
| PAO1/pME6032*-orf172* | Overexpression of *orf172* using pME6032 plasmid in PAO1 | This lab |
| PAO1/pME6032*-orf173* | Overexpression of *orf173* using pME6032 plasmid in PAO1 | This lab |
| PAO1/pME6032*-orf174* | Overexpression of *orf174* using pME6032 plasmid in PAO1 | This lab |
| PAO1/pME6032*-orf175* | Overexpression of *orf175* using pME6032 plasmid in PAO1 | This lab |
| PAO1/pUCP24*-dap2* | Overexpression of *dap2* using pUCP24 plasmid in PAO1 | This study |
| PAO1/pUCP24-*dap2*(E97A) | Overexpression of *dap2*(E97A) using pUCP24 plasmid in PAO1 | This study |
| PAO1/pUCP24*-dap2*(E99A) | Overexpression of *dap2*(E99A) using pUCP24 plasmid in PAO1 | This study |
| PAO1/*p*-*dap1* | Overexpression of *dap1* using pHERD20T plasmid in PAO1 | This lab |
| PAO1/*p*-*dap2* | Overexpression of *dap2* using pHERD20T plasmid in PAO1 | This study |
| PAO1/*p*-*dap2*(V52K) | Overexpression of *dap2*^V52K^ using pHERD20T plasmid in PAO1 | This study |
| PAO1/*p*-*dap2*(A46K) | Overexpression of *dap2*^A46K^ using pHERD20T plasmid in PAO1 | This study |
| PAO1/*p*-*dap2*(N79A) | Overexpression of *dap2*^N79A^ using pHERD20T plasmid in PAO1 | This study |
| PAO1/*p*-*dap2*(E97A) | Overexpression of *dap2*^E97A^ using pHERD20T plasmid in PAO1 | This study |
| PAO1/*p*-*dap2*(E99A) | Overexpression of *dap2*^E99A^ using pHERD20T plasmid in PAO1 | This study |
| PAO1/*p*-*dap1dap2* | Overexpression of *dap1dap2* using pHERD20T plasmid in PAO1 | This study |
| PAO1/*p-hnh* | Overexpression of *hnh* using pHERD20T plasmid in PAO1 | This lab |
| ***E. coli*** |  |  |
| DH5α | *F – φ80lacZ ΔM15 Δ(lacZYA-argF) U169 recA1 endA1 hsdR17(rk–, mk+) phoA supE44 thi-1 gyrA96 relA1 tonA* | Stratagene |
| BL21（DE3） | *F-, ompT, hsdSB (rBB - mB -), gal, dcm (DE3)* | Stratagene |
| BTH101 | *F-, cya-99, araD139, galE15, galK16, rpsL1 ( Strr ), hsdＲ2, mcrA1, mcrB1* | (McCarthy et al, 2017) |
| XL1-BlueMRF' Kan H | Host strain for propagating pBT and pTRG recombinants;*Δ(mcrA)183Δ(mcrCB-hsdSMR-mrr)173 endA1 supE44 thi-1 recA1 gyrA96 relA1 lac* [F' *proAB lacI^q^ Z ΔM15* Tn*5*(Kn^r^)] | Agilent |
| XL1-BlueMRF' Kan R | Report strain of BacterioMatch II two-hybrid system;*Δ(mcrA)183Δ(mcrCB-hsdSMR-mrr)173 endA1 supE44 thi-1 recA1 gyrA96 relA1 lac* [F' *laqI^q^ HIS3 aadA* (Kn^r^)] | Agilent |
| **Phage** |  |  |
| PaoP5 | *P. aeruginosa* phage | (Shen *et al*, 2016) |
| PaoP5Δ*dap2* | Knockout *dap2* in PaoP5 | This study |
| PaoP5Δ*dap1*Δ*dap2* | Knockout *dap1*and *dap2* in PaoP5 | This study |
| PaoP5Δ*orf014* | Knockout *orf014* in PaoP5 | (Shen *et al.*, 2016) |
| **Plasmids** |  |  |
| pME6032 | Plasmid for over-expression of gene in PAO1 and PaoP5 | (Heeb *et al*, 2002) |
| pMMB67EH-Flag | pMMB67EH vector with Flag tag coding sequence | (Chen et al., 2020) |
| pHERDB20T | Plasmid for over-expression of gene in PaoP5; Gm^r^ | (Zhou *et al*, 2022) |
| pUCP24 | Plasmid for over-expression of gene in PaoP5 | (Lasarte-Monterrubio *et al*, 2022) |
| pUT18C | Plasmid for bacterial-two hybrid assays | (McCarthy *et al.*, 2017) |
| pKT25 | Plasmid for bacterial-two hybrid assays | (McCarthy *et al.*, 2017) |
| pET28a | Plasmid with His tag for protein expression and purification | (Wang *et al*, 2020) |
| pTCPLS | Plasmid with CRISPPR-cas9 for gene knockout in PaoP5 | (Yang *et al*, 2023) |
| pMS402 | Plasmid with lux-based reporters for Luminescence screening assays | (Duan *et al*, 2003) |
| pBT | p15A origin of replication, *lac-UV5* promoter, λ cI open reading fram; Cm^r^ | Agilent |
| pTRG | ColE1 origin of replication, lpp promoter, lac-UV5 promoter, RNAPα open reading frame; Tc^r^ | Agilent |
| pBT-LGF2 | Interaction control plasmid encoding the dimerization domain (40 amino acid residues) of the Gal4 transcriptional activator protein; Cm^r^ | Agilent |
| pTRG-Gal11^p^ | Interaction control plasmid encoding a domain (90 amino acid residues) of the mutant form of the Gal11 protein; Tc^r^ | Agilent |
| pKD-*exsA* | pMS402 containing *exsA* promoter region; Kn^r^, Tmp^r^ | (Duan *et al.*, 2003) |
| pKD-*exsC* | pMS402 containing *exsCEB* promoter region; Kn^r^, Tmp^r^ | (Duan *et al.*, 2003) |
| pKD-*exoS* | pMS402 containing *exoS* promoter region; Kn^r^, Tmp^r^ | (Duan *et al.*, 2003) |
| pKD-*exoT* | pMS402 containing *exoT* promoter region; Kn^r^, Tmp^r^ | (Duan *et al.*, 2003) |
| pKD-*exoY* | pMS402 containing *exoY* promoter region; Kn^r^, Tmp^r^ | (Duan *et al.*, 2003) |
| mini-CTX*-exsA*(*p_exsC_*)-FLAG | Mini-CTX-*lacZ* containing the entire *exsA* gene driven by *exsCEBA* promoter fused with FLAG tag at C-terminal; Tc^r^ | This lab |
| pGEX-6p-1 | Expression vector with N-terminal GST tag; Amp^r^ | This lab |
| pGEX-6p-1-*dap2* | pGEX6p-1 derivative for expression of *dap2* | This study |
| pGEX-6p-1-*dap2*(V52K) | pGEX6p-1 derivative for expression of *dap2*(V52K) | This study |
| pGEX-6p-1-*dap2*(A64K) | pGEX6p-1 derivative for expression of *dap2*(A64K) | This study |
| pGEX-6p-1-*dap2*(N79A) | pGEX6p-1 derivative for expression of *dap2*(N79A) | This study |
| pGEX-6p-1-*dap2*(V52KA64KN79A) | pGEX6p-1 derivative for expression of *dap2*(V52KA64KN79A) | This study |
| pGEX-6p-1-*dap2*(E97A) | pGEX6p-1 derivative for expression of *dap2* (E97A) | This study |
| pGEX-6p-1-*dap2*(E99A) | pGEX6p-1 derivative for expression of *dap2* (E99A) | This study |
| pGEX-6p-1-*dap2*(E97AE99A) | pGEX6p-1 derivative for expression of *dap2* (E97AE99A) | This study |
| pET28a-*exsA* | Protein expression construct, the entire gene of *exsA* cloned in pET28a vector | This study |
| pET28a-*dap1* | Protein expression construct, the entire gene of *dap1* cloned in pET28a vector | (Shen *et al.*, 2016) |
| pET28a-*hnh* | Protein expression construct, the entire gene of *hnh* cloned in pET28a vector | This study |
| pET28a-*lon* | Protein expression construct, the entire gene of *lon* cloned in pET28a vector | This study |
| pBT-*dap2* | pBT plasmid containing the entire *dap2* gene | This study |
| pTRG-*exsA* | pTRG plasmid containing the entire *exsA* gene | This study |
| pUT18C-*dap2* | *dap2* cloned into pUT18C for complementation | This study |
| pUT18C-*dap2*(E97A) | *dap2*(E97A) cloned into pUT18C for complementation | This study |
| pUT18C-*dap2*(E99A) | *dap2*(E99A) cloned into pUT18C for complementation | This study |
| pKT25-*dap1* | *dap1* cloned into pKT25 for complementation | This study |
| pKT25-*hnh* | *hnh* cloned into pKT25 for complementation | This study |
| pKT25-*lon* | *lon* cloned into pKT25 for complementation | This study |

**References:**

Duan K, Dammel C, Stein J, Rabin H, Surette MG (2003) Modulation of Pseudomonas aeruginosa gene expression by host microflora through interspecies communication. *Mol Microbiol* 50: 1477-1491

Heeb S, Blumer C, Haas D (2002) Regulatory RNA as mediator in GacA/RsmA-dependent global control of exoproduct formation in Pseudomonas fluorescens CHA0. *J Bacteriol* 184: 1046-1056

Lasarte-Monterrubio C, Fraile-Ribot PA, Vázquez-Ucha JC, Cabot G, Guijarro-Sánchez P, Alonso-García I, Rumbo-Feal S, Galán-Sánchez F, Beceiro A, Arca-Suárez J *et al* (2022) Activity of cefiderocol, imipenem/relebactam, cefepime/taniborbactam and cefepime/zidebactam against ceftolozane/tazobactam- and ceftazidime/avibactam-resistant Pseudomonas aeruginosa. *J Antimicrob Chemother* 77: 2809-2815

McCarthy RR, Mazon-Moya MJ, Moscoso JA, Hao Y, Lam JS, Bordi C, Mostowy S, Filloux A (2017) Cyclic-di-GMP regulates lipopolysaccharide modification and contributes to Pseudomonas aeruginosa immune evasion. *Nat Microbiol* 2: 17027

Shen M, Le S, Jin X, Li G, Tan Y, Li M, Zhao X, Shen W, Yang Y, Wang J *et al* (2016) Characterization and Comparative Genomic Analyses of Pseudomonas aeruginosa Phage PaoP5: New Members Assigned to PAK_P1-like Viruses. *Sci Rep* 6: 34067

Wang T, Qi Y, Wang Z, Zhao J, Ji L, Li J, Cai Z, Yang L, Wu M, Liang H (2020) Coordinated regulation of anthranilate metabolism and bacterial virulence by the GntR family regulator MpaR in Pseudomonas aeruginosa. *Mol Microbiol* 114: 857-869

Yang L, Wang J, Lu S, Zhong Y, Xiong K, Liu X, Liu B, Wang X, Wang P, Le S (2023) Temperature-dependent carrier state mediated by H-NS promotes the long-term coexistence of Y. pestis and a phage in soil. *PLoS pathog* 19: e1011470

Zhou D, Huang G, Xu G, Xiang L, Huang S, Chen X, Zhang Y, Wang D (2022) CRISPRi-Mediated Gene Suppression Reveals Putative Reverse Transcriptase Gene PA0715 to Be a Global Regulator of Pseudomonas aeruginosa. *Infect Drug Resist* 15: 7577-7599
