# Supplementary material for: Simultaneous inhibition of bacterial virulence and anti-phage defense systems by synergistic bacteriophage counter-defense proteins
Source: EMBO J. 2026 Mar 18;45(8):2756–84. doi: 10.1038/s44318-026-00740-0 (PMC13083879; doi:10.1038/s44318-026-00740-0)
Supplement: Supplementary file 2 — Table EV2 [file 44318_2026_740_MOESM2_ESM.docx]

**Table EV2. Primers used in this study**

| **Primers** | **Sequence (5’–3’)** |
| --- | --- |
| **Overexpression of**  **genes in PAO1** |  |
| pHERDB20T-*orf004*-F | GCGAATTCGAGCTCGGTACatgtacgacaaggctcaag |
| pHERDB20T-*orf004*-R | AGCTTGCATGCCTGCAttagttttcattctcttcctccattt |
| pHERDB20T-*orf004*(E97A)-R | AGCTTGCATGCCTGCAttagtttGcattctcttcctccattt |
| pHERDB20T-*orf004*(E99A)-R | AGCTTGCATGCCTGCAttagttttcattcGcttcctccattt |
| pHERDB20T-*orf003004*-F | AAACGATGGCGATTGCGatgtacgacaaggctcaagt |
| pHERDB20T-*orf003004*-R | CGACGGCCAGTGCCAttagaccctcccgaac |
| pME6032-*orf004*-F | CAAgaattcatgtacgacaaggctcaa |
| pME6032-*orf004*-R | CCGctcgagttagttttcattctcttc |
| pME6032-*orf001*-F | ATActcgagCTAGCGGTAGGCCTTGAGC |
| pME6032-*orf001*-R | tatcgatgcatgccatggtacttaccacgccgatggtc |
| pME6032-*orf003*-F | AACgaattcatgaaaactaaagagatcgatg |
| pME6032-*orf003*-R | TATaagcttTTAGACCCTCCCGAACGT |
| pME6032-*orf005*-F | TACgaattcAtgcgggtgtactaccga |
| pME6032-*orf005*-R | TGCctcgagtcaattgtattcctcctc |
| pME6032-*orf006*-F | GCGgaattcatgtttaagaagctgttc |
| pME6032-*orf006*-R | TATctcgag tcagtaccctgtcaagct |
| pME6032-*orf007*-F | ACAgaattcatggaacaagtagggctt |
| pME6032-*orf007*-R | AAActcgagtcagtcctcctctcctgt |
| pME6032-*orf008*-F | CACgaattcatggagataatcccaatg |
| pME6032-*orf008*-R | ATActcgagctacttgttccatggtgc |
| pME6032-*orf009*-F | GGCgaattcatgaactctaattctattgca |
| pME6032-*orf009*-R | TATctcgagttactcccctccttttcg |
| pME6032-*orf010*-F | GCCgaattcatgatttctctggatgtg |
| pME6032-*orf010*-R | AGTctcgagtcagcacatcacaaccca |
| pME6032-*orf011*-F | gataacaatttcacacaggaaacagatgttcaagaaactcgtaacc |
| pME6032-*orf011*-R | tatcgatgcatgccatggtactcacagcatttcctcccg |
| pME6032-*orf012*-F | GCCgaattcatgttgacatgtgaagag |
| pME6032-*orf012*-R | CTActcgagctaaccctcctcttgatc |
| pME6032-*orf013*-F | GCCgaattcatgtatacagttgaggttcca |
| pME6032-*orf013*-R | TGCctcgagtcacatgtcaacatacgg |
| pME6032-*orf131*-F | ATAgaattcatgaacagcaaactccgc |
| pME6032-*orf131*-R | ATCctcgagttaaaacacctccagtcc |
| pME6032-*orf132*-F | TAGgagctcatgaaagaatattttaaaa |
| pME6032-*orf132*-R | TGTctcgagttatactacctcctcaaa |
| pME6032-*orf133*-F | TCTgagctcatggaacaaactattgat |
| pME6032-*orf133*-R | TTActcgagttagtctacctcttctagga |
| pME6032-*orf134*-F | TAGgagctcAtggatatgttcgataaa |
| pME6032-*orf134*-R | CTTctcgagttaggcttcatatgcttc |
| pME6032-*orf135*-F | AAAgagctcAtgaagcctaatgttgta |
| pME6032-*orf135*-R | ATCctcgagttattctgtgtcaagcct |
| pME6032-*orf136*-F | AAAgagctcatggccactacagagaaa |
| pME6032-*orf136*-R | TACctcgagttaaaagtccaatgtgtt |
| pME6032-*orf137*-F | GGTgagctcatgataaaagattttgtaaaa |
| pME6032-*orf137*-R | GGTctcgagtcacatcttgctgaagtt |
| pME6032-*orf138*-F | AAAgagctcAtgaaagcacagcctaaagaa |
| pME6032-*orf138*-R | AAActcgagtcaccactccatgccgac |
| pME6032-*orf139*-F | AAAgagctcatgtccgcgactaacaagtgc |
| pME6032-*orf139*-R | AAActcgagtcaagctaggggcccggg |
| pME6032-*orf140*-F | AAAgagctcAtgactagccgctggaaa |
| pME6032-*orf140*-R | ACActcgagttacagaaggtcgtctgg |
| pME6032-*orf141*-F | TCAgagctcAtgcaattcaatatatcgaag |
| pME6032-*orf141*-R | TGTctcgagttattttcgccactctgc |
| pME6032-*orf142*-F | AAAgagctcatgaagattgacaacggt |
| pME6032-*orf142*-R | ACActcgagttagatcgcctcgtattc |
| pME6032-*orf143*-F | TTTgagctcatgctacatagtgaagcc |
| pME6032-*orf143*-R | TTActcgagtcaacccttattttcgtt |
| pME6032-*orf144*-F | ACCgagctcAtgaaagctctttgtaatcat |
| pME6032-*orf144*-R | TTGctcgagttatgctgtgcggaaaga |
| pME6032-*orf145*-F | ATAgagctcAtgcaagcttggaacgta |
| pME6032-*orf145*-R | AATctcgagttactcggcctcctccac |
| pME6032-*orf146*-F | TTTgagctcAtggataacctgtccgtt |
| pME6032-*orf146*-R | TTTctcgagtcatgccgtttttacttc |
| pME6032-*orf147*-F | taaGAGCTCatgaccgtcagagtagga |
| pME6032-*orf147*-R | atcCTCGAGTCAAGCGTTTATTTCACTa |
| pME6032-*orf148*-F | taaGAGCTCAtgcaaatgtttttcaaa |
| pME6032-*orf148*-R | atcCTCGAGTTACTTAGCCTCCTTTTT |
| pME6032-*orf149*-F | gagGAGCTCAtggctaagtctgttagt |
| pME6032-*orf149*-R | ccgCTCGAGTTAATCGAATTTATACTC |
| pME6032-*orf150*-F | ataGAGCTCAtggcacgttctatcaag |
| pME6032-*orf150*-R | attCTCGAGTTACATATCGCTACTGAAGTC |
| pME6032-*orf151*-F | atagaattcAtggctaggcgtcaacggctc |
| pME6032-*orf151*-R | ataCTCGAGCTAGGCGGCCTGTCCGCC |
| pME6032-*orf152*-F | agaGAGCTCAtgaacttcactctcaatct |
| pME6032-*orf152*-R | aatCTCGAGTTAACCCTTAGCAATGAG |
| pME6032-*orf153*-F | taaGAGCTCAtggacgaatctgtttac |
| pME6032-*orf153*-R | tatCTCGAGTTACCGGTCTCCTTTGTA |
| pME6032-*orf154*-F | AACgagctcATGCGACAGCTTAATAAATC |
| pME6032-v*orf154*-R | ACTctcgagTCACTGGTAACGCTCGAT |
| pME6032-*orf155*-F | TAAgagctcATGAAAGCTGTAGCTCAGTT |
| pME6032-*orf155*-R | ATActcgagTTAGATGGCCGATTTAGC |
| pME6032-*orf156*-F | GAAgagctcATGAAAATTTCCGCTATCC |
| pME6032-*orf156*-R | AATctcgagTCATTTCCCTTGAGCCTC |
| pME6032-*orf158*-F | CTCgaattcATGTCGATCTTAGCCTTAACT |
| pME6032-*orf158*-R | TATctcgagTCATGGCAGTTCATCCTC |
| pME6032-*orf159*-F | TTAgaattcATGATTGCACCCCGTCTT |
| pME6032-*orf159*-R | TATctcgagTTACAGAAAAGCTAGGTTGGG |
| pME6032-*orf160*-F | TTAgaattcATGGCAATCGGTGATAGC |
| pME6032-*orf160*-R | TATctcgagTCAATACCAAACTTCACGCA |
| pME6032-*orf161*-F | TTAgagctcATGAATCGCACCACCCTG |
| pME6032-*orf161*-R | ATActcgagCTAGCGGTAGGCCTTGAGC |
| pME6032-*orf162*-F | ATAgagctcATGATCCTCGTTCACGGC |
| pME6032-*orf162*-R | TTTctcgagTCACTCCTCCTCTTTGTCTTT |
| pME6032-*orf166*-F | CGGgaattcATGAGCCAGATTAAAATCC |
| pME6032-*orf166*-R | TATctcgagTCAGGCTGCCAGTTCGAG |
| pME6032-*orf167*-F | CACgagctcATGAAACGCCAATATACTG |
| pME6032-*orf167*-R | TCTctcgagTTACGGTTTCCATCCTGA |
| pME6032-*orf168*-F | CTGgagctcATGAATATCCTGTTTATCCA |
| pME6032-*orf169*-F | TTAgagctcATGCACCTGAAGACACTA |
| pME6032-*orf169*-R | TATctcgagTCATTTGACGATTTCTCC |
| pME6032-*orf170*-F | CTGgagctcATGAAGAGCAAGTATGCA |
| pME6032-*orf170*-R | GCCctcgagTTATTTGCGTTTGTTCAT |
| pME6032-*orf171*-F | TTAgagctcATGGCAAAGGCAAATCTG |
| pME6032-*orf171*-R | TATctcgagTTAGGGTAAACGGAGCCC |
| pME6032-*orf172*-F | TAGgagctcATGCAAGCAACCTACCAA |
| pME6032-*orf172*-R | CGCctcgagTTACTTGCGATACTTCTTAA |
| pME6032-*orf173*-F | CCAgagctcATGAATAACTCGCCTGTAGT |
| pME6032-*orf173*-R | AATctcgagTTAGCGACGAAGGGTTGT |
| pME6032-*orf174*-F | TTAgagctcATGAACGCCACTCTGGCA |
| pME6032-*orf174*-R | ATTctcgagTTAGTGATGGCTGCGGATGC |
| pME6032-*orf175*-F | TTAgagctcATGCTTATGCAATTCGTG |
| pME6032-*orf175*-R | TATctcgagTCATTTTGCAATGAGAATTG |
| pUCP24- *orf004*-F | ACGAATTCGAGCTCGGTACatgtacgacaaggctca |
| pUCP24- *orf004*-R | TGCCTGCAGGTCGACTttagttttcattctcttcctcc |
| pUCP24- *orf004*(E97A)-R | TGCCTGCAGGTCGACTttagttGcattctcttcctcc |
| pUCP24- *orf004*(E99A)-R | TGCCTGCAGGTCGACTttagttttcattcGcttcctcc |
|  |  |
| **Knock out phage genes** |  |
| *004-*G1F | TAGTacacttcatgagtgtgacac |
| *004*-G1R | AAACgtgtcacactcatgaagtgt |
| Δ*004*-LA-F | GGTCTGACAGCTCGAGagtctaatgagaactgcttcc |
| Δ*004*-LA-R | agttttcatgaagccgtttccgc |
| Δ*004*-RA-F | aacggcttcatgaaaactaaagagatcgatgtc |
| Δ*004*-RA-R | TTTTTTTGGCGCGCCattgctcagacattagaccc |
| Δ*003004*-LA-F | GGTCTGACAGCTCGAGagtctaatgagaactgcttc |
| Δ*003004*-LA-R | ctcccgagaagccgtttccgctc |
| Δ*003004*-RA-F | acggcttctcgggagggtctaatgtc |
| Δ*003004*-RA-R | TTTTTTTGGCGCGCctccctagtggcgtagttc |
| Δ*003*-RO | gcgatggttgtatagattcacc |
| Δ003-LO  Δ004-RO | agacccctgaagagaacatc  gcttttgatgcacccgatag |
| Δ*004*-LO | tccttacgcccgaaaaag |
|  |  |
| **3-AT** |  |
| pBT-004-F | ATAgcggccgcAATGTACGACAAGGCCTCAA |
| pBT-004-R | CACgaattcTTAGTTTTCATTCTCTTCCTCCATT |
| pTRG-*exsA*-F | ATAgcggccgcAATGCAAGGAGCCAAATAT |
| pTRG-*exsA*-R | CACgaattcTCAGTTATTTTTAGCCCGGC |
|  |  |
| **BATCH** |  |
| pUT18C-*004*-F | TGGAACGCCACTGCAatgtacgacaaggctcaagt |
| pUT18C-*004*-R | AGTGCACCATATTACTTAGTTATATttagttttcattctcttcctccattt |
| pUT18C-*004*(E97A)-R | AGTGCACCATATTACTTAGTTATATttagtttGcattctcttcctccattt |
| pUT18C-*004*(E99A)-R | AGTGCACCATATTACTTAGTTATATttagttttcattcGcttcctccattt |
| pKT25-*003*-F | ACGCGGCGGGCTGCAatgaaaactaaagagatcgatgt |
| pKT25-*003*-R | AGTGAATTCTTACTTACTTAGGTACttagaccctcccgaacgtt |
| pKT25-*050*-F | ACGCGGCGGGCTGCAatgaagctgtgccctcgc |
| pKT25-*050*-R | AGTGAATTCTTACTTACTTAGGTACctaaactacaatctcatatccttcaatacc |
| pKT25-*lon*-F | ACGCGGCGGGCTGCAatgaaaacactcgtcgaattg |
| pKT25-*lon*-R | AGTGAATTCTTACTTACTTAGGTACctaatgcgtgctaattcgc |
|  |  |
| **RT-qRCR** |  |
| 16s-F | CAAAAGCTACTGAGCTAGAGTACG |
| 16s-R | TAAGATCTCAAGGATCCCAACGGCT |
| *exsA*-qpcr-F | GGAGAATCCTCTATGCCCATCA |
| *exsA*-qpcr-R | CTCTGGGTGAAATAGGACTGACTG |
| *exsC*-qpcr-F | TGGCACCGTTTCGATCTGCA |
| *exsC*-qpcr-R | GCCAAGGTCGCCTCGAAGCATT |
| *exoS*-qpcr-F | AGGAGCTGGATGCGGGACAAA |
| *exoS*-qpcr-R | CCACGGGTGCCACGGAAAGT |
| *exoT*-qpcr-F | CGCGAAATCGCCGTCCAA |
| *exoT*-qpcr-R | AGCCCGAAGTGCTCCACCAG |
| *exoY*-qpcr-F | GCATGGCAGTGGTGGTCTCG |
| *exoY*-qpcr-R | CCATAGAATCCGTCCTCGCTCA |
| *hemH*-qpcr-F | TGGAAAGCGTGCGTCCGTACCTG |
| *hemH*-qpcr-R | GGGCTGACGTTGCGGCTGTTCT |
| *hfp*-qpcr-F | CACGCAAGCAGGCGGAGAGC |
| *hfp*-qpcr-R | TGGCAGCGGAAAGCGAATAG |
| *pscF*-qpcr-F | GAATACCCTCGATACCGTGG |
| *pscF*-qpcr-R | GTTGATGTTGTAGATGACCG |
| *pcrV*-qpcr-F | CCGAAGCAGAGCGGGGAA |
| *pcrV* -qpcr-R | CCGAGTTGTAGCGGGAGC |
| *orf004*-qpcr-F | AGTGCTACTGTGGGTTGG |
| *orf004*-qpcr-R | TGGCCTCTTTGAGTTCTT |
| *orf053*-qpcr-F | CGGTTCTGTCGGTGGTCT |
| *orf053*-qpcr-R | TCAACAGGCTCGTCGTCT |
|  |  |
| **Promoter** |  |
| *excC* promoter-F | GCCGTCTCCGCGCGGGAGGA |
| *excC* promoter-R | GGGGGCGCCTCCTAAAGCTC |
|  |  |
| **Protein expression and purification** |  |
| pEGX-6p-1-*orf004*-F | CTGTTCCAGGGGCCCatgtacgacaaggctcaa |
| pEGX-6p-1-*orf004*-R | CGTCAGTCAGTCACGATGCttagttttcattctcttcctccatttct |
| pEGX-6p-1-*orf004*(E97A)-R | CGTCAGTCAGTCACGATGCttagtttGcattctcttcctccatttct |
| pEGX-6p-1-*orf004*(E99A)-R | CGTCAGTCAGTCACGATGCttagttttcattcGcttcctccatttct |
|  |  |
| **Point mutation** |  |
| *orf004-*DTB-F | cgggtgtactaccgagactta |
| *orf004*-DTB-R | cttgcttggctagggttgct |
| *orf004*(V52K)-F | acctacgacttccgaaggaAAgacttccatttcgtca |
| *orf004*(V52K)-R | tgacgaaatggaagtcTTtccttcggaagtcgtaggt |
| *orf004*(A64K)-F | gagaagaaagtgaccgAAtacacacttcatgagtgtga |
| *orf004*(A64K)-R | tcacactcatgaagtgtgtaTTcggtcactttcttctc |
| *orf004*(N79A)-F | gtcctactacagagctaGCtggcacctacacgcttca |
| *orf004*(N79A)-R | tgaagcgtgtaggtgccaGCtagctctgtagtaggac |
| *orf004*(E97A)-F | tgcagaaatggaggaagcgaatgaaaactaaagagat |
| *orf004*(E97A)-R | atctctttagttttcattcGcttcctccatttctgca |
| *orf004*(E99A)-F | aatggaggaagagaatgCaaactaaagagatcgatgt |
| *orf004*(E99A)-R | acatcgatctctttagtttGcattctcttcctccatt |
| *orf004*(E97AE99A)-F | ggaagCgaatGCAaactaaagagatcgatgtc |
| *orf004* (E97AE99A)-R | gttTGCattcGcttcctccatttctgcaa |
