# Supplementary material for: Simultaneous inhibition of bacterial virulence and anti-phage defense systems by synergistic bacteriophage counter-defense proteins
Source: EMBO J. 2026 Mar 18;45(8):2756–84. doi: 10.1038/s44318-026-00740-0 (PMC13083879; doi:10.1038/s44318-026-00740-0)
Supplement: Supplementary file 20 — Expanded View Figures [file 44318_2026_740_MOESM20_ESM.pdf]

Expanded View Figures

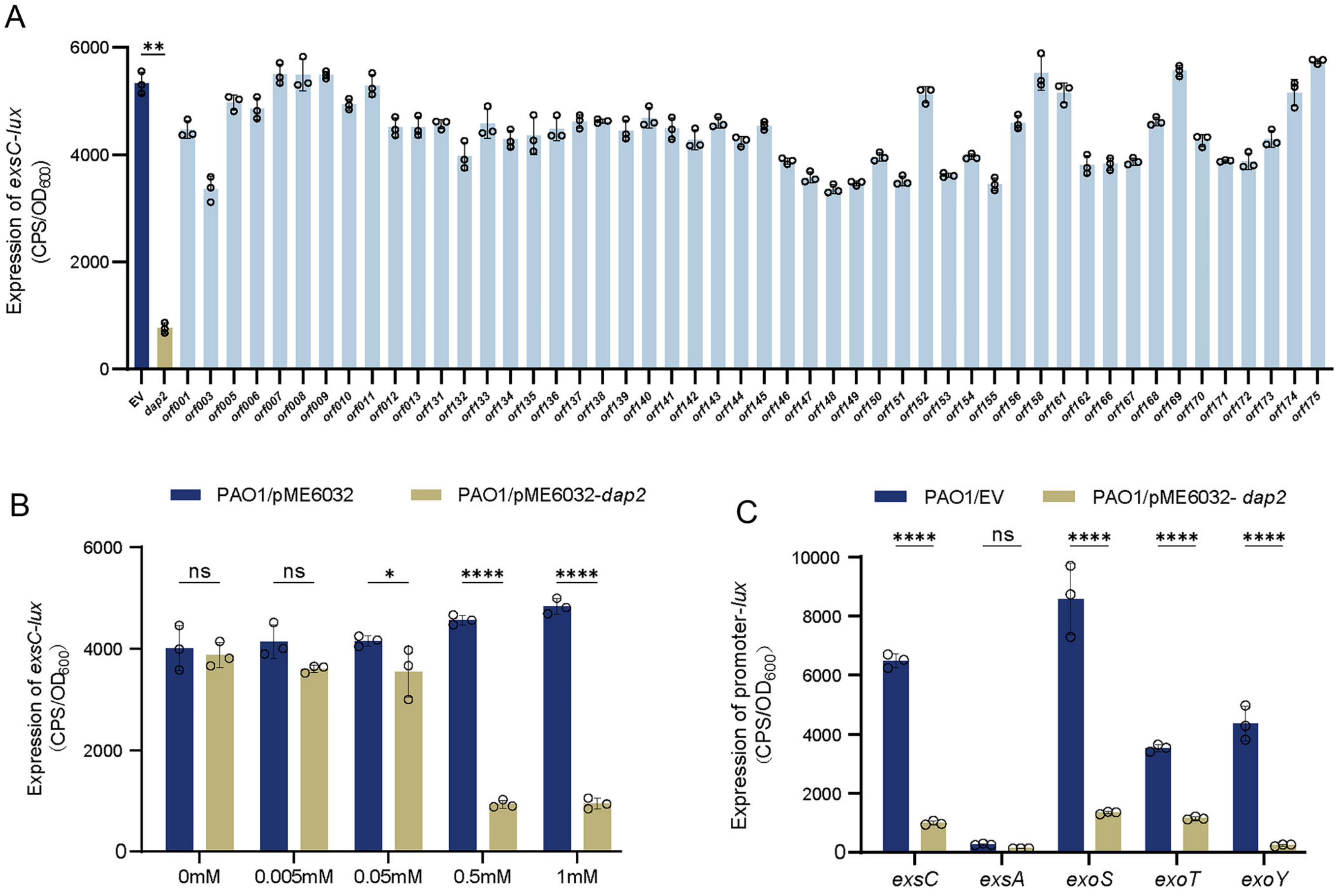

Figure EV1. Overexpression of *dap2* inhibited the activity of T3SS-related genes.

(**A**) The promoter activity of *exsC* was assessed in strains carrying either *exsC-lux* or 51 hypothetical ORFs derived from PaoP5. These constructs were individually expressed using the pME6032 vector and cultured in LB medium supplemented with 5 mM EGTA, 20 mM $MgCl_2$, and 0.5 mM IPTG. Promoter activity measurements were performed following 6 h of cultivation. Error bars represent the mean ± SD of three independent experiments. Statistical significance was determined using a two-sided Student's t-test (PAO1/EV vs PAO1/-*dap2*: $P < 0.0001$). EV, empty vector. **$P < 0.01$. (**B**) The expression of *exsC* in PAO1/pME6032 and PAO1/pME6032-*dap2* cultured in T3SS inducing medium supplemented with different concentrations of IPTG. Error bars represent the mean ± SD of three independent experiments. Two-way ANOVA was used to calculate *p* value (PAO1/EV vs PAO1/-*dap2*: 0 mM IPTG, $P = 0.9760$; 0.005 mM IPTG, $P = 0.0994$; 0.05 mM IPTG, $P = 0.0480$; 0.5 mM IPTG, $P < 0.0001$; 1 mM IPTG, $P < 0.0001$). ns, not significant, *$P < 0.05$, ****$P < 0.0001$. (**C**) The promoter activities of pKD-*exsA*, pKD-*exsC*, pKD-*exoS*, pKD-*exoT*, and pKD-*exoY* were comparatively analyzed in PAO1/EV and PAO1-pME6032-*dap2* strains under T3SS-inducing conditions supplemented with 0.5 mM IPTG. Following 6-hour cultivation, transcriptional activity measurements revealed differential expression patterns. (**A–C**) Error bars represent the mean ± SD of three independent experiments. Statistical significance was determined using a two-sided Student's *t*-test (PAO1/EV vs PAO1/-*dap2*: *exsC*, $P < 0.0001$; *exsA*, $P = 0.9983$; *exoS*, $P < 0.0001$; *exoT*, $P < 0.0001$; *exoY*, $P < 0.0001$). EV, empty vector. ns, not significant. ****$P < 0.0001$. Source data are available online for this figure.

© The Author(s)

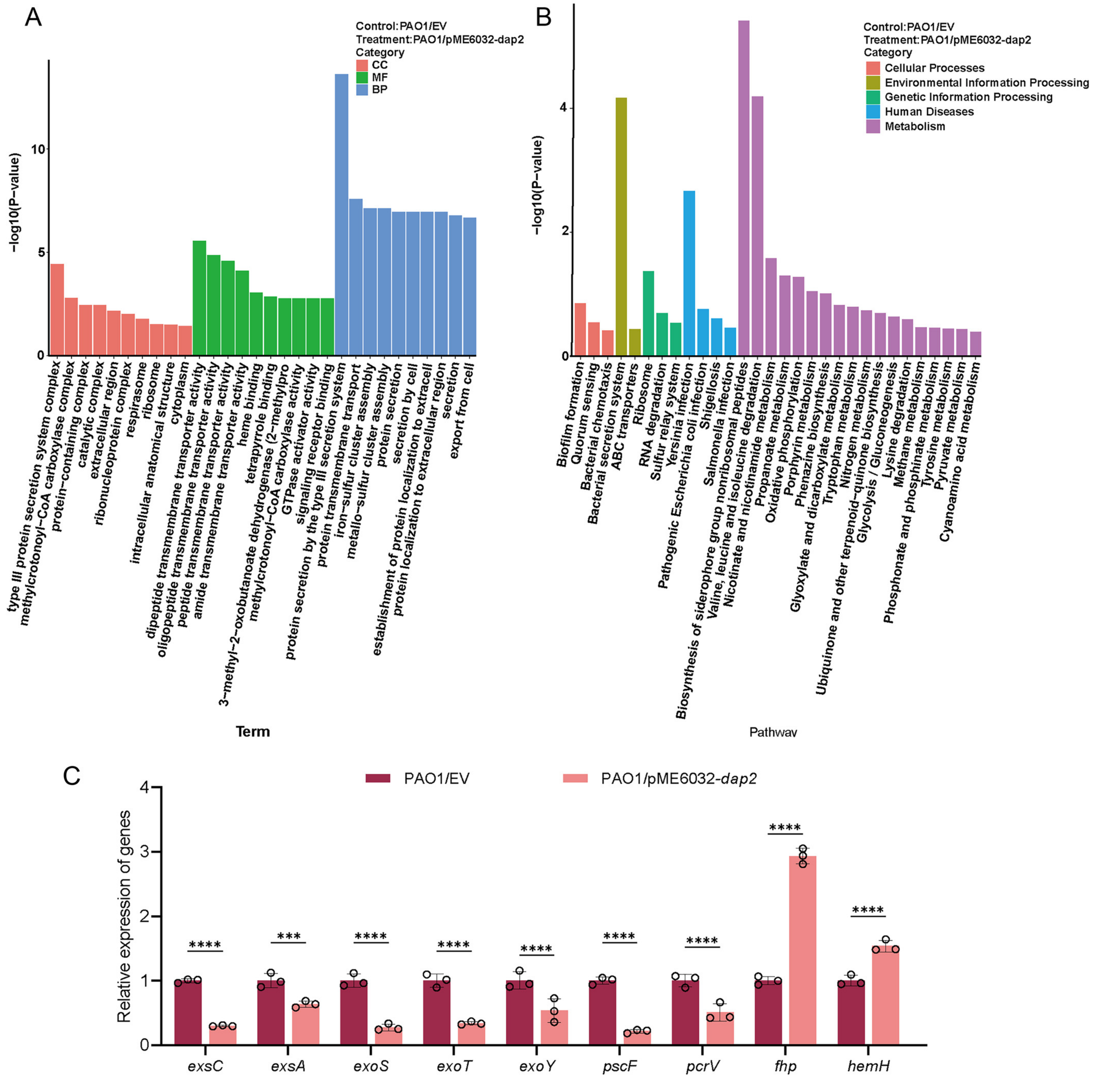

Figure EV2. RNA-seq analysis between PAO1 and PAO1/pME6032-*dap2*.

(**A**) The GO enrichment of the differentially expressed genes in PAO1/EV and PAO1/pME6032-*dap2* strains, which is classified according to molecular function (MF), biological process (BP), and cellular component (CC), and the top 10 enriched GO was shown. (**B**) The DEGs are classified based on the KEGG analysis, and the top 30 enriched pathways, including Quorum Sensing and biofilm formation, are displayed. (**C**) Comparative qRT-PCR analysis of target gene expression in PAO1/EV versus PAO1/pME6032-*dap2* strains under T3SS-inducing conditions. Quantitative data are presented as mean ± SD of three independent experiments. Statistical significance was determined by two-way ANOVA (PAO1/EV vs PAO1/-*dap2*: *exsC*, $P < 0.0001$; *exsA*, $P = 0.0002$; *exoS*, $P < 0.0001$; *exoT*, $P < 0.0001$; *exoY*, $P < 0.0001$; *pscF*, $P < 0.0001$; *pcrV*, $P < 0.0001$; *fhp*, $P < 0.0001$; *hemH*, $P < 0.0001$). EV, empty vector. ***$P < 0.001$, ****$P < 0.0001$. Source data are available online for this figure.

 © The Author(s)

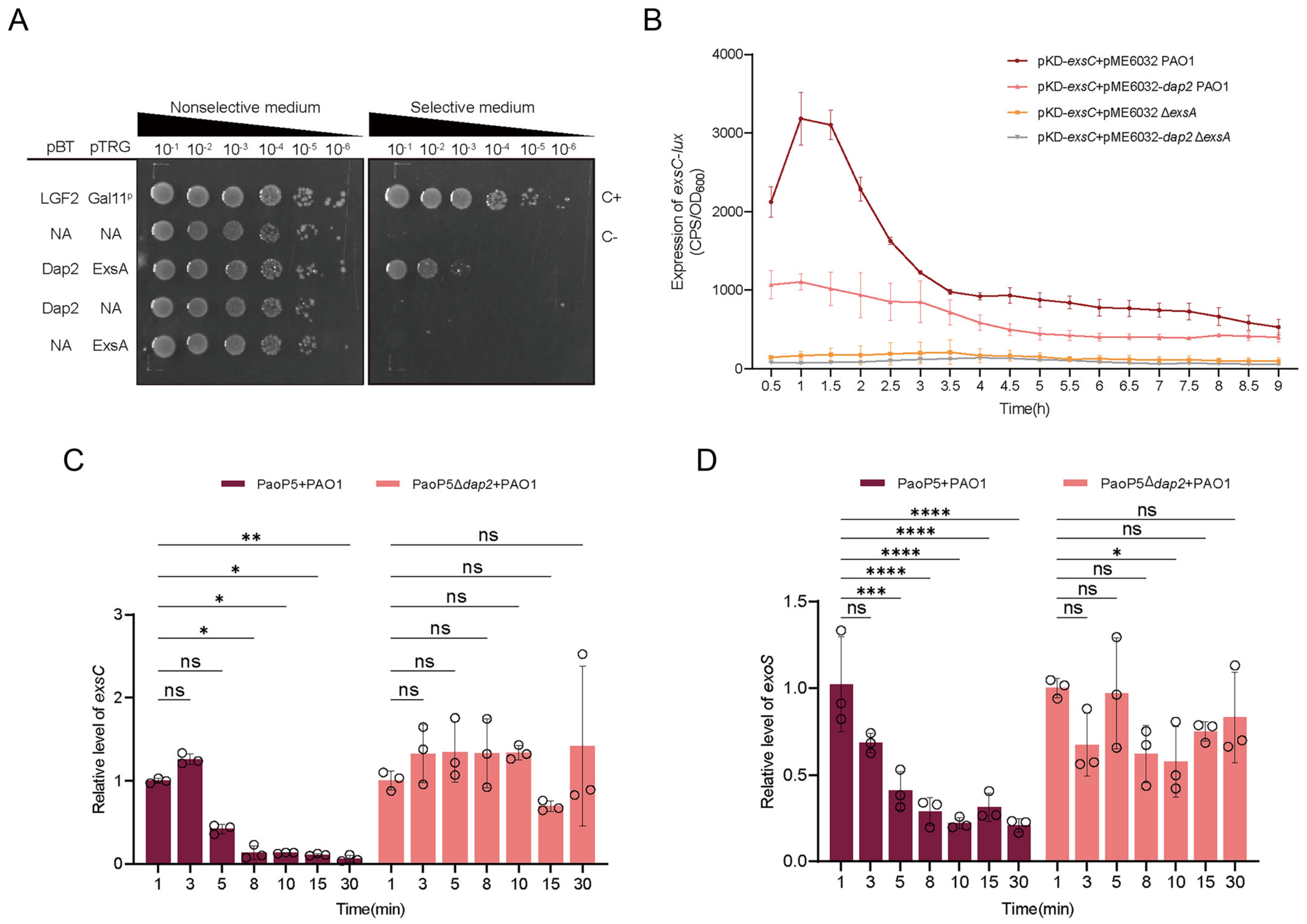

Figure EV3. Verification of the interaction between Dap2 and ExsA in vitro and in vivo.

(**A**) *E. coli* two-hybrid assay reveals an interaction between Dap2 and ExsA. The recombinant strains harboring different plasmids were separately streaked on nonselective and dual-selective media. The strain expressing LGF2 and GAI11P was used as a positive control. (**B**) Expression of *dap2* did not suppress *exsC* activity in Δ*exsA* mutant strain. *P. aeruginosa* carrying pKD-*exsC* with pME6032-*dap2* was cultivated in T3SS-inducing medium. The promoter activity was detected at specified time points. Error bars represent mean ± SD of three biological replicates. (**C**, **D**) Expression of exsC and exoS during phage PaoP5 and PaoP5Δ*dap2* infection at the given time points. Error bars represent the mean ± SD of three independent experiments. Statistically significant variations were demonstrated by two-way ANOVA (PaoP5 + PAO1:0 min vs 3 min, $P = 0.8997$; 0 min vs 5 min, $P = 0.1769$; 0 min vs 8 min, $P = 0.0135$; 0 min vs 10 min, $P = 0.0129$; 0 min vs 15 min, $P = 0.0105$; 0 min vs 30 min, $P = 0.0065$; PaoP5Δ*dap2* + PAO1: 0 min vs 3 min, $P = 0.7734$; 0 min vs 5 min, $P = 0.7226$; 0 min vs 8 min, $P = 0.7645$; 0 min vs 10 min, $P = 0.7462$; 0 min vs 15 min, $P = 0.8022$; 0 min vs 30 min, $P = 0.5398$). EV, empty vector. ns, not significant, $*P < 0.05$, $**P < 0.01$, $***P < 0.001$, $****P < 0.0001$. Source data are available online for this figure.

© The Author(s)

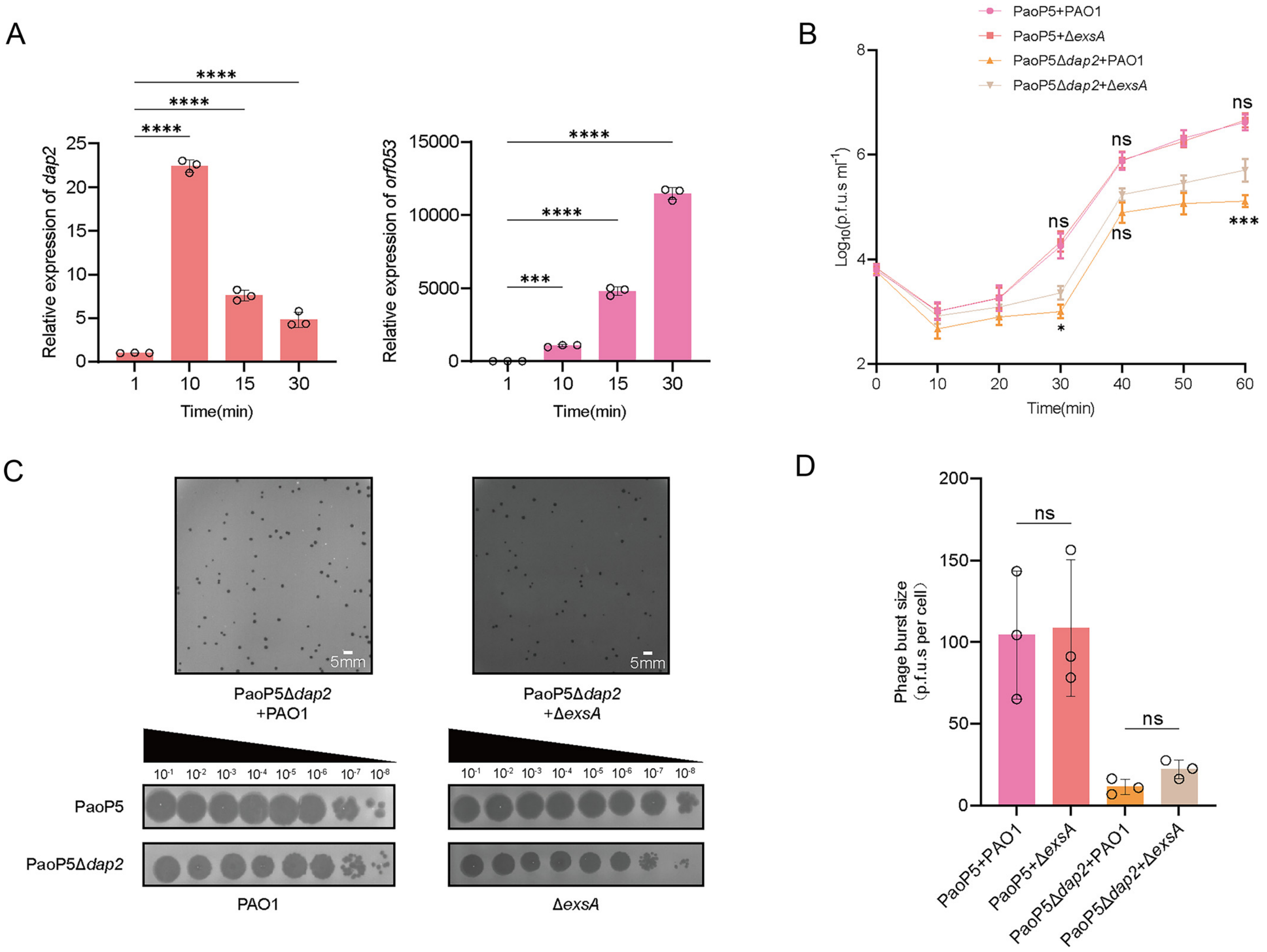

Figure EV4. Dap2 is an early-expressed gene and deletion of *exsA* did not affect phage plaque formation.

(**A**) qRT-PCR analysis of *dap2* and *orf53* expression at 1-, 10-, 15-, and 30-min after phage infection. *dap2* was expressed immediately after entering the host and the expression decreased after 10 min. Error bars represent the mean ± SD of three independent experiments. Statistically significance was assessed by one-way ANOVA (*dap2*:1 min vs 10 min, $P < 0.0001$; 1 min vs 15 min, $P < 0.0001$; 1 min vs 30 min, $P < 0.0001$; *orf053*: 1 min vs 10 min, $P = 0.0008$; 1 min vs 15 min, $P < 0.0001$; 1 min vs 30 min, $P < 0.0001$). ***$P < 0.001$, ****$P < 0.0001$. (**B**) The one-step growth curve of phage PaoP5 or PaoP5Δ*dap2* infecting PAO1 or Δ*exsA*. Error bars represent the mean ± SD of three independent experiments. Statistical significance was determined using two-way ANOVA (PaoP5 + PAO1 vs PaoP5+Δ*exsA*: 30 min, $P = 0.0782$; 40 min, $P < 0.0001$; 60 min, $P < 0.0001$; PaoP5Δ*dap2* + PAO1 vs PaoP5Δ*dap2*+Δ*exsA*: 30 min, $P = 0.0782$;40 min, $P < 0.0001$; 60 min, $P < 0.0001$). ns, not significant, *$P < 0.05$, ***$P < 0.001$. (**C**) Phage PaoP5Δ*dap2* was mixed with PAO1 or Δ*exsA*, and used double-layer agar plates to observe the plaque number and size. Data are representative of three independent replicates. (**D**) The burst size of wild-type PaoP5 infecting PAO1 and PAO1Δ*exsA* was 104.35 ± 27.67 PFU/cell and 108.70 ± 29.65 PFU/cell, respectively. In contrast, the burst size of PaoP5Δ*dap2* infecting PAO1 and PAO1Δ*exsA* was significantly reduced to 11.36 ± 3.89 PFU/cell and 22.08 ± 3.99 PFU/cell, respectively. Error bars represent the mean ± SD of three independent experiments. Statistical significance was determined using one-way ANOVA by Dunnett's multiple comparison test (PaoP5 + PAO1 vs PaoP5+Δ*exsA*, $P = 0.9972$; PaoP5Δ*dap2* + PAO1 vs PaoP5Δ*dap2*+Δ*exsA*, $P = 0.9613$). ns, not significant, *$P < 0.05$. Source data are available online for this figure.

© The Author(s)

A

Accession #	Molecular weight	Cover percentage	Peptides	m/z	PTM
tr|Q9I5F9|Q9I5F9_PSEAE	88575	81%	K.KGVAMTGELTLTGQVLPIGGVR.E	1099.1	
			K.GVAM(+15.99)TGELTLTGQVLPIGGVR.E	1043	Oxidation(M)
			K.VLFVC(+57.02)TANTLDSIPGPLLDR.M	1101.1	Carbamidomethylation
			K.E(-18.01)SAEIAYSYIGSHLKK.Y	593.3	Pyro-glu from E
			K.ELLPLNPLYSEELKN(+.98)YLNR.F	773.7	Deamidation(NQ)
			R.Q(-17.03)KIFELILPEANR.G	777	Pyro-glu from

B

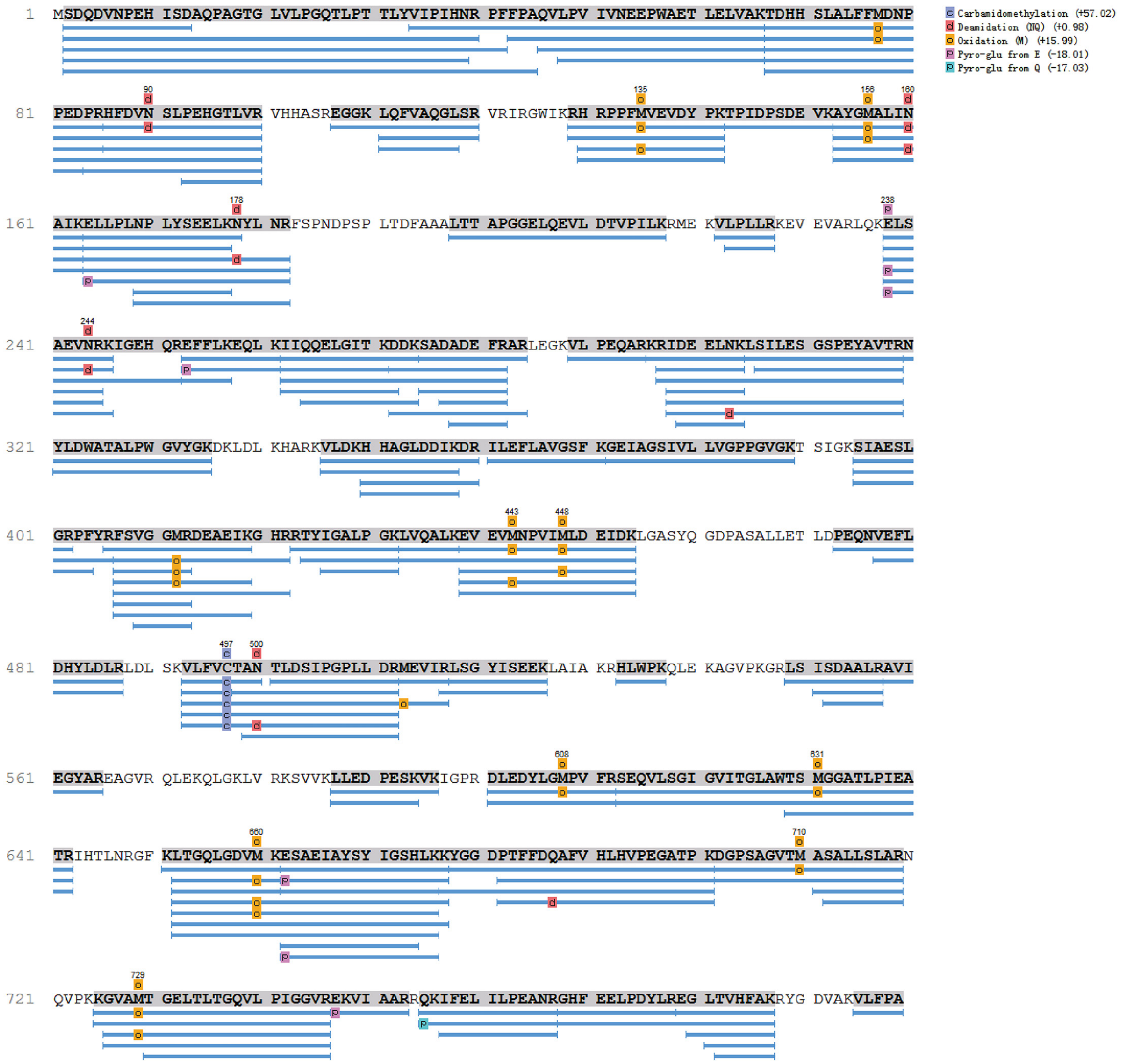

Figure EV5. Identification of Lon protease that interacts with Dap2.

(**A**) The specific protein bands identified in the Co-IP experiment were excised and subjected to mass spectrometry (MS) analysis. The MS results confirmed Lon as a novel interaction partner of Dap2, as indicated by the peptide matches and corresponding scores listed in the search results. (**B**) The coverage map displays peptide detection across the amino acid sequence of Lon, with peaks indicating identified regions.

 © The Author(s)

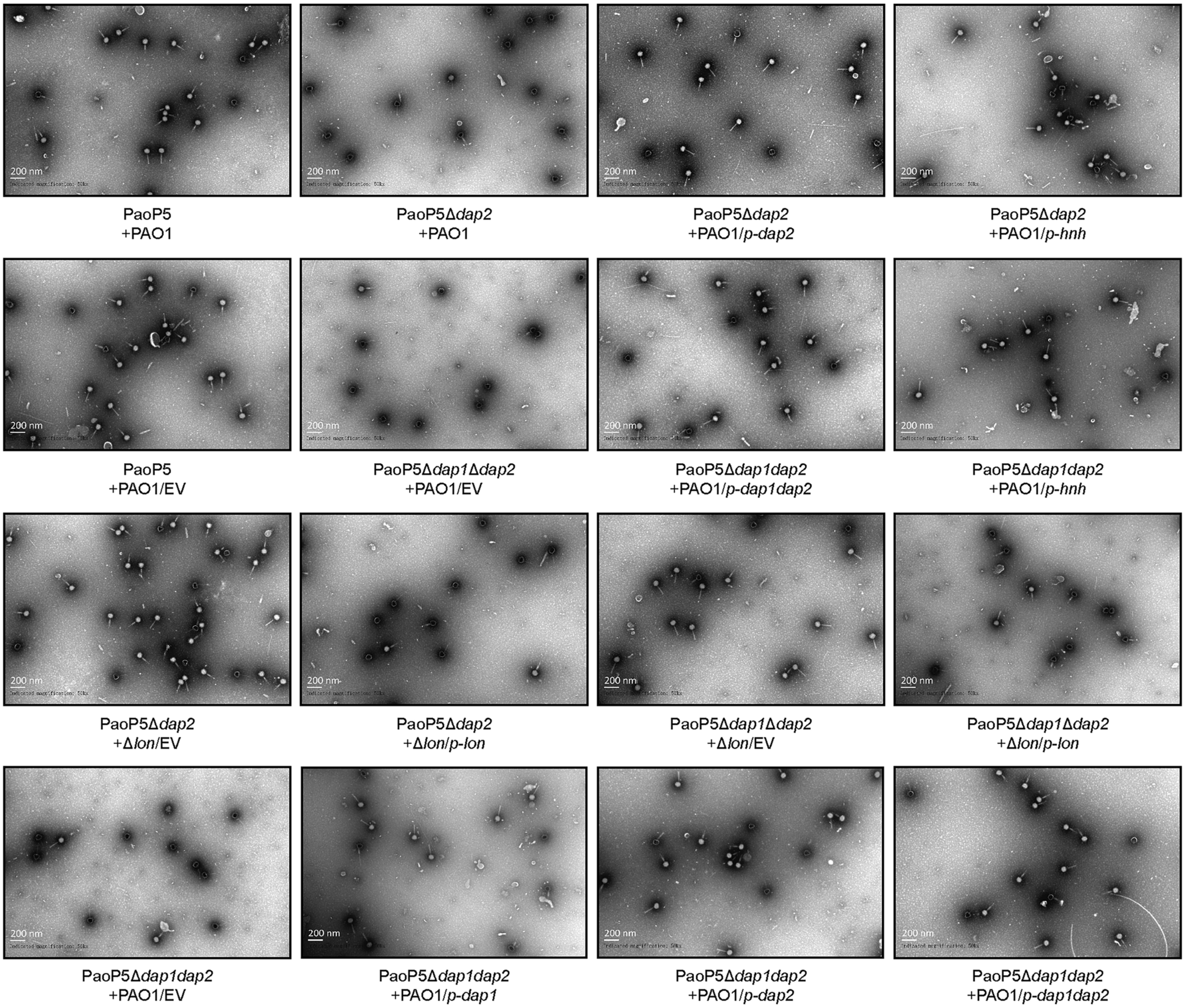

Figure EV6. Representative transmission electron micrographs of negatively stained phages produced in PAO1, PAO1/*p-hnh*, PAO1/*p-dap2*, PAO1/*p-dap2dap2*, Δ*lon*, and Δ*lon*/*p-lon*.

The empty capsids are black, and phages with white heads are packaged with the genomes. Source data are available online for this figure.

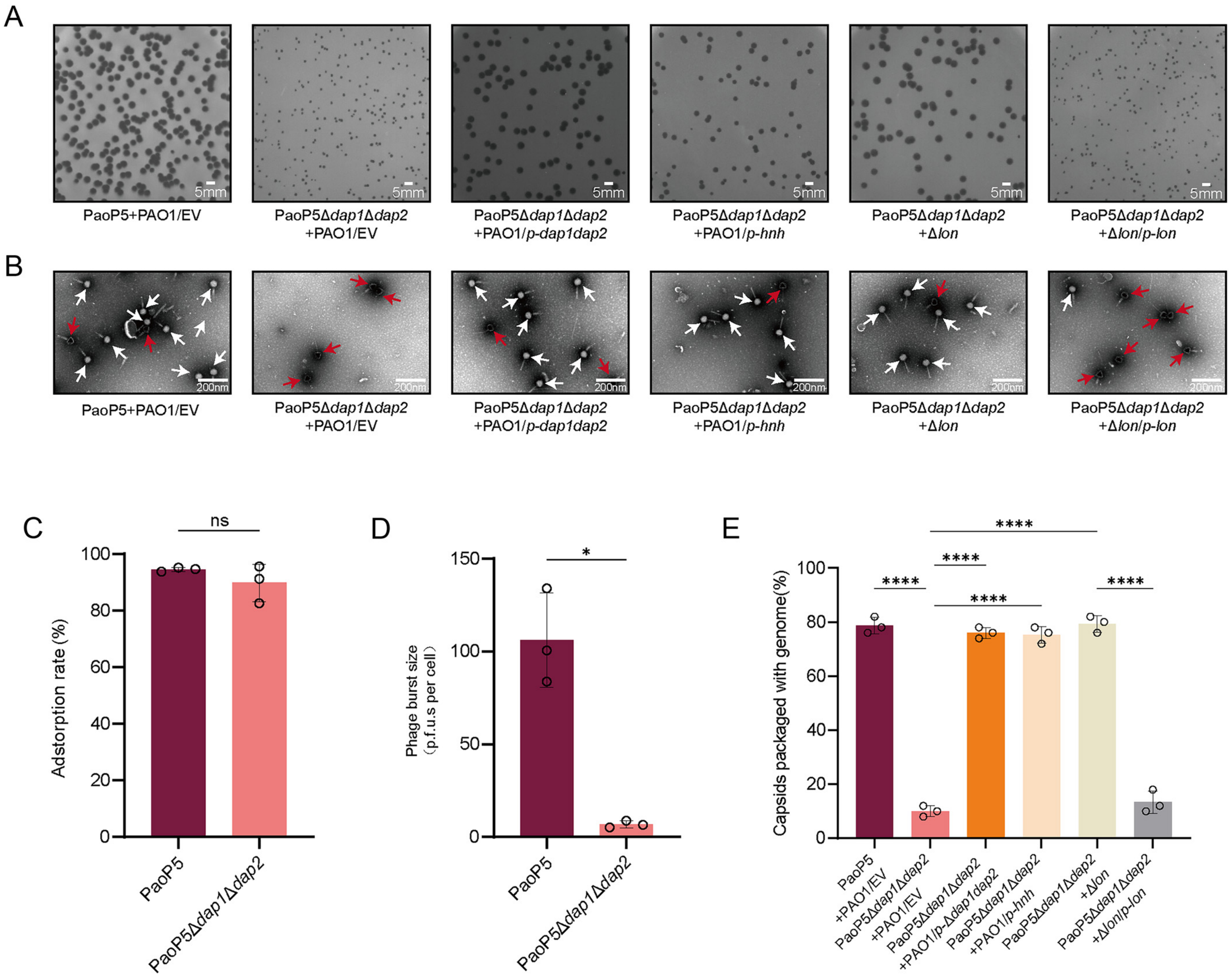

Figure EV7. Impact of Lon and HNH on phage PaoP5Δ*dap1*Δ*dap2*.

(**A**) The plaques of PaoP5 or PaoP5Δ*dap1*Δ*dap2* infecting PAO1/EV, PAO1/*p-dap1dap2*, PAO1/*p-hnh*, Δ*lon*, and Δ*lon*/*p-lon*. (**B**) Representative TEM of negatively stained phages produced in PAO1/EV, PAO1/*p-dap1dap2*, PAO1/*p-hnh*, Δ*lon*, and Δ*lon*/*p-lon*. The red arrows indicate the empty capsids and the phages in which the genome is packaged are indicated by the white arrows. (**C**) Both PaoP5Δ*dap1*Δ*dap2* and PaoP5 phages adsorbed to PAO1 efficiently. Error bars represent the mean ± SD of three independent experiments. Statistical significance was determined by an unpaired, two-tailed Student's *t* test (PaoP5 vs PaoP5Δ*dap1*Δ*dap2*: $P = 0.3680$). ns, not significant. (**D**) The burst size of phage PaoP5 or PaoP5Δ*dap1*Δ*dap2* infecting PAO1. respectively. Error bars represent the mean ± SD of three independent experiments. Statistical significance was determined using a two-sided Student's t-test (PaoP5 vs PaoP5Δ*dap1*Δ*dap2*: $P = 0.0229$). *$P < 0.05$. (**E**) Phages were cultured in the indicated strains, and the percentage of capsids packaged with genomes was calculated from three biological repeats. Three biological repeats were performed, and 50 particles were counted for the presence or absence of the genome. Error bars represent the mean ± SD of three independent experiments. Significance versus the control group was assessed by one-way ANOVA with Dunnett's multiple comparisons test. *P* value was calculated based on one-way ANOVA Dunnett's multiple comparison test (PaoP5Δ*dap1*Δ*dap2* + PAO1 vs PaoP5 + PAO1, $P < 0.0001$; PaoP5Δ*dap1*Δ*dap2* + PAO1 vs PaoP5Δ*dap1*Δ*dap2* + PAO1/*p- dap1dap2*, $P < 0.0001$; PaoP5Δ*dap1*Δ*dap2* + PAO1 vs PaoP5Δ*dap1*Δ*dap2* + PAO1/*p-hnh*, $P < 0.0001$; PaoP5Δ*dap1*Δ*dap2* + PAO1 vs PaoP5Δ*dap1*Δ*dap2*+Δ*lon*, $P < 0.0001$; PaoP5Δ*dap1*Δ*dap2*+ Δ*lon* vs PaoP5Δ*dap1*Δ*dap2*+Δ*lon*/*p-lon*, $P < 0.0001$). EV, empty vector. ****$P < 0.0001$. Source data are available online for this figure.

© The Author(s)

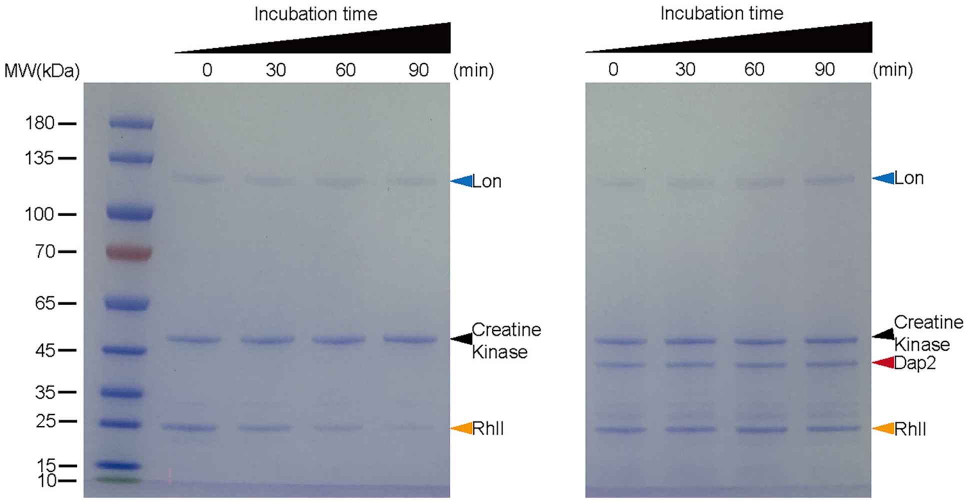

Figure EV8. In vitro proteolysis assays showed that Lon effectively degraded RhlI, and this degradation was suppressed upon the addition of Dap2.

Protein samples collected at designated time points were analyzed using 12% SDS–PAGE and visualized by Coomassie blue staining. All experiments were independently repeated at least three times with consistent outcomes, and representative results are presented.

© The Author(s)
